# Longitudinal Follow-Up on Cardiopulmonary Exercise Capacity Related to Cardio-Metabolic Risk Factors in Children With Renal Transplants

**DOI:** 10.3389/fspor.2021.688383

**Published:** 2021-08-16

**Authors:** Susanne Westphal Ladfors, Ebba Bergdahl, Oli Hermannsson, Julius Kristjansson, Tina Linnér, Per Brandström, Sverker Hansson, Frida Dangardt

**Affiliations:** ^1^Pediatric Nephrology, The Queen Silvia Children's Hospital, Gothenburg, Sweden; ^2^Department of Molecular and Clinical Medicine, Institute of Medicine, Sahlgrenska Academy, University of Gothenburg, Gothenburg, Sweden; ^3^Pediatric Clinical Physiology, The Queen Silvia Children's Hospital, Gothenburg, Sweden; ^4^Department of Pediatrics, Institute of Clinical Sciences, Sahlgrenska Academy, University of Gothenburg, Gothenburg, Sweden

**Keywords:** kidney, renal transplantation, children, pediatric, cardiopulmonary exercise capacity, blood pressure, ambulatory blood pressure monitoring, cardiopulmonary exercise test

## Abstract

**Background:** Children with chronic kidney disease, including those treated with kidney transplantation (KT), have an increased risk of cardiovascular disease. The aim of this study was to examine the cardiopulmonary exercise capacity after KT compared to matched controls, to relate the results to physical activity, blood pressure and biochemical findings and to follow exercise capacity over time.

**Methods:** Patients with KT (*n* = 38, age 7.7–18 years), with a mean time from transplantation of 3.7 years (0.9–13.0) and mean time in dialysis 0.8 years, were examined at inclusion and annually for up to three years. Healthy controls (*n* = 17, age 7.3–18.6 years) were examined once. All subjects underwent a cardiopulmonary exercise test, resting blood pressure measurement, anthropometry and activity assessment. Patients also underwent echocardiography, dual-energy X-ray absorptiometry (DXA), 24-h ambulatory BP measurements (ABPM), assessment of glomerular filtration rate (GFR) and blood sampling annually.

**Results:** As compared to healthy controls, KT patients showed decreased exercise capacity measured both as VO_2peak_ (34.5 vs. 43.9 ml/kg/min, *p* < 0.001) and maximal load (2.6 vs. 3.5 W/kg, *p* < 0.0001), similarly as when results were converted to z-scores. No significant difference was found in weight, but the KT patients were shorter and had higher BMI z-score than controls, as well as increased resting SBP and DBP z-scores. The patient or parent reported physical activity was significantly lower in the KT group compared to controls (*p* < 0.001) In the combined group, the major determinants for exercise capacity z-scores were activity score and BMI z-score (β = 0.79, *p* < 0.0001 and β = −0.38, *p* = 0.007, respectively). Within the KT group, low exercise capacity was associated with high fat mass index (FMI), low activity score, low GFR and high blood lipids. In the multivariate analysis FMI and low GFR remained predictors of low exercise capacity. The longitudinal data for the KT patients showed no change in exercise capacity z-scores over time.

**Conclusion:** Patients with KT showed decreased exercise capacity and increased BP as compared to healthy controls. Exercise capacity was associated to GFR, physical activity, FMI and blood lipids. It did not improve during follow-up.

## Introduction

Kidney transplantation (KT) is an established treatment method and the treatment of choice for children as well as adults with chronic kidney disease and end-stage renal disease (ESRD) as it offers a dramatically better quality of life, improved survival and reduction in overall morbidity. Earlier transplantation, pre-emptive transplantation and improved routines during the last decades have further improved the prognosis (McDonald and Craig, 2004; Francis et al., 2020).

However, chronic kidney disease and kidney transplantation are associated with an increased risk of cardiovascular complications, and the mortality risk of cardiovascular disease (CVD) is still higher in the KT-group than in healthy children (Foster et al., 2011). CVD is the second most common cause of death among renal transplanted children and adolescents, even with new treatment strategies and improved cardiovascular management (Smith et al., 2007; NAPRTCS, 2014; Chesnaye et al., 2018).

Physical exercise has been proven to prevent and/or delay development of cardiovascular complications and promote weight control in kidney transplanted children to a similar degree as in the healthy pediatric population (Krasnoff et al., 2006). Low cardiorespiratory capacity is an independent risk factor for death from CVD in the adult population (Kodama et al., 2009). The most common risk factors for CVD following renal transplantation in childhood are the common denominators of the metabolic syndrome; hypertension, hyperlipidemia, hyperinsulinemia and obesity (Wilson et al., 2010). These are also associated with increased left ventricular mass (Wilson et al., 2010), as well as with decreased cardiorespiratory capacity (Armstrong et al., 2006).

Lubrano et al. (2012) showed that physically active children with KT could reach a fitness level comparable with sedentary healthy controls and a better level than transplanted sedentary children. Children with KT could benefit from physical activity, increasing their VO_2peak_ and time in exercise assessed by cardiopulmonary exercise test (CPET), if 3–5 h weekly exercise is achieved.

There is little or no information in the literature on the longitudinal evolvement of the exercise capacity in children after KT. As children with KT often are recommended to limit sports participation (Wolf et al., 2016), and parental overprotection commonly occurs (Hullmann et al., 2010), CPET could offer a controlled and safe way to demonstrate to KT patients and their families that an individual with KT can exercise safely. An annual exercise test may offer encouragement to increase physical activity and be used for monitoring of fitness level.

The aim of this study was to examine the cardiopulmonary exercise capacity in children in a stable situation after KT compared to matched healthy controls, to relate the results to physical activity, blood pressure, cardiac function and biochemical findings and to follow exercise capacity in patients over time.

## Materials and Methods

### Study Subjects

We aimed to enroll all eligible patients with a functioning graft about 1 year after kidney transplant performed before the age of 18 years at our regional center, consecutively at their annual visit during the years 2013–2018. Exclusion criterium was the inability to perform a CPET, hence children below 7 years of age were excluded from enrolment. Healthy controls with similar age and sex ratio as the KT children were recruited from schools in the area for a cross-sectional comparison at one time point. With this sample size and enrolment ratio, the probability was 90% that the study would detect a difference in VO2 between groups of 9 mL/kg/min, at a two-sided 0.05 significance level. The study was conducted in accordance to the Declaration of Helsinki, and ethical approval was obtained from the local ethical committee. All participants and guardians received both written and oral information, and informed consent were collected.

Patients with KT were examined at inclusion and annually for up to 3 years for longitudinal data. Healthy controls were examined once for cross-sectional comparison between groups.

### Anthropometry

Height was measured to the nearest 0.1 cm using a stadiometer and weight was measured to the nearest 0.1 kg on a digital scale. Body mass index (BMI) was calculated as weight/height^2^, z-scores were calculated from the WHO reference criteria (Rodd et al., 2014).

### Cardiopulmonary Exercise Test

As part of their annual clinical visit, all children with KT monitored at The Queen Silvia Children's Hospital perform a CPET.

The CPET comprises a 1-min-step-protocol cycle test on an electrically braked bicycle ergometer with breath-by-breath analysis of VO_2_ (oxygen uptake) and VCO_2_ (carbon dioxide excretion) (Oxycon Pro, Erich Jaeger GmbH, Hoechberg, Germany). A 12-lead ECG was collected simultaneously by Cardiolex EC sense (Cardiolex Medical AB, Stockholm, Sweden) and monitored continuously. The test protocol consisted of a 3-min warm-up phase of pedaling without load, then incremental increase of load by 5–20 W/min, starting at 20 W, until peak exercise was reached, followed by 2-min rest (Ten Harkel et al., 2011). Increment of load during the test (5–20 W/min) was chosen individually based on weight and the self-reported physical capacity and activity, with an aim of reaching peak exercise at 10–12 min. Respiratory exchange ratio (RER) was calculated by dividing produced CO_2_ by consumed O_2_. Peak exercise was determined as the 30 s before termination. The test was considered sufficient if the subject reached an exhaustion of >17 on the Borg-scale for perceived exertion, preferably when RER was ≥1.1. For some of the youngest subjects, this was not achieved. Values for VO_2_, VCO_2_ and HR were collected as 30-s average at peak exercise. Z-scores were calculated using normal values from Ten Harkel et al. (2011), where a similar CPET protocol have been used.

### Activity Score

Physical activity was assessed by a semi-structured interview of the patients and accompanying parents including questions on leisure time activity, school transportation, recess time activity and sports participation during the last year, based on the international physical activity questionnaire (IPAQ) (Arvidsson et al., 2005). Subjects were categorized as follows: 1: no regular physical activity, 2: moderate to vigorous physical activity 1–2 h per week and 3: moderate to vigorous physical activity 3 h or more per week.

### Blood Pressure

Ambulatory blood pressure monitoring (ABPM) was performed using a portable oscillometric device (SpaceLabs Monitor 90207; SpaceLabs Medical Inc., Redmond, Washington, USA) for all patients at baseline and at annual follow-up. Cuff size was selected according to the upper arm circumference. Blood pressure was measured for 24 h and daytime systolic and diastolic BP as well as nighttime systolic and diastolic BP were analyzed according to Elke Wühl et al. (2002). All measurements were normalized according to standard deviation scores (SDS). Non-dipping was defined as a decline in mean nighttime systolic or diastolic blood pressure with <10% of the corresponding daytime value.

Resting systolic (SBP) and diastolic blood pressure (DBP) was measured manually by an experienced examiner, with the subject in supine position after a 10 min rest.

### Echocardiography

KT patients underwent a complete transthoracic cardiac ultrasound in subcostal, suprasternal notch, and right parasternal views, according to pediatric echocardiogram guidelines (Lai et al., 2006), using General Electric Vivid E9 (GE Health Medical, Horten, Norway). Examinations were performed by one experienced investigator to minimize the measurement variance within the group, and the post-processing analysis software EchoPAC 11 (GE Health Medical, Horten, Norway) was used for standardized measurements. Left ventricular diameter in diastole (LVd), interventricular septum thickness in diastole (IVSd) and posterior wall thickness in diastole (PWd) was measured in 2D-guided M-mode images from parasternal short axis views at the midpapillary level. Left ventricular mass (LVM) was estimated using the Devereaux formula (Devereux et al., 1986), which allows for calculation of left ventricular mass index (LVMI) using the formula *LVMI* = *LVM*(*g*)/*height*(*m*)^2,7^ (Foster et al., 2016).

### Biochemistry

Blood samples were collected in the patients with KT, at baseline and then annually at follow-up. Fasting blood samples were collected and prepared for central analysis. Serum lipids (triglycerides, total cholesterol, high-density (HDL) and low-density (LDL) lipoprotein cholesterol) were analyzed using an enzymatic calorimetric method and serum creatinine with an enzymatic method, all measured photometrically (Cobas 6000 Roche Diagnostica Scandinavia AB) at an accredited lab. The inter assay variation did not exceed 5% in any of the analyses.

#### Glomerular Filtration Rate

GFR was assessed based on the plasma disappearance curve after single bolus injection of a glomerular tracer (^51^Cr-EDTA) with a minimal dose of 0.074 MBq/kg and maximal dose 3.7 MBq. The clearance was calculated from the injected dose divided by the area under the curve, using the bi-exponential method (Sapirstein et al., 1955). The exact injected dose was determined by weighing the syringe before and after the injection on a high precision analytic balance. Accurately timed blood samples were drawn at five, 15, 60, 90, and 120 min after the injection and the blood samples and the standard were measured in a well counter. The GFR was calculated using the Bröchner-Mortensen correction (Bröchner-Mortensen et al., 1974).

### Criteria for Metabolic Syndrome

Metabolic syndrome was considered present when three of the following five criteria were fulfilled: BMI z-score > 2, hypertension (systolic or diastolic blood pressure > 95th or on antihypertensive treatment), serum glucose > 5.6 mmol/L, triglycerides > 1.7 mmol/L and HDL cholesterol < 1.03 mmol/L (Wilson et al., 2010).

### Dual-Energy X-Ray Absorptiometry

All children with KT underwent a DXA scan for assessment of body composition, using Lunar iDXA (GE Lunar Corp., Madison, WI, USA). The normative healthy reference database, which uses sex, weight, ethnicity- and age-specific reference data, is provided by the DXA machine manufacturer LUNAR (GE Medical Systems Lunar). Analyses of fat mass (FM, kg) and lean body mass (LBM, kg) were made. Fat mass index (FMI) was calculated as FM divided by height squared (kg/m^2^), lean mass index (LMI) was calculated as LBM divided by height squared (kg/m^2^).

### Statistical Analysis

The distribution of continuous variables is given as mean, SD, median, minimum, and maximum and of categorical variables as number and percentages. For comparison between two groups with respect to continuous variables, the Mann–Whitney U-test was used, and for ordered categorical variables, Mantel-Haenszel test was used. Correlation was described by using Spearman correlation coefficients. Associations between VO2 peak/kg/min z-score and FMI, GFR, activity score, LMI and lipids were investigated using linear regression. The correlations between VO2peak and maximal load and lean body mass (LMI) were tested after log-transformation to ensure normal distribution, and to permit comparison with Sethna et al. (2009). Stepwise linear regression was performed for selection of a significant multivariable model. Parameter estimates (β), 95% confidence intervals (CI), *p*-values and *R*^2^ were presented from these analyses. The assumptions of normality and homoscedasticity of residuals were reviewed in diagnostic plots and found fulfilled. Changes of continuous variables over time were tested using paired *t*-test. All the tests were two-tailed and conducted at the 5% significance level. Analyses were performed using the SAS® version 9.4 software (SAS Institute Inc, Cary, NC, USA).

## Results

Forty-two children above seven years of age with KT and functioning grafts, who were considered able to perform a CPET were included. Four patients were later excluded due to inability to complete the exercise test. The 38 KT children and adolescents (18F/20M), median age of 13.6 years (range 7.7–18), with a mean time from transplantation of 3.7 years (0.9–13.0) and mean time in dialysis (*n* = 26) 0.8 years (range 4 days−5.4 years) were examined at inclusion and annually for up to 3 years. Seventeen matched healthy controls (*n* = 17, 7F/10M) with a median age of 11.7 years (range 7.3–18.6) were examined once (Table 1).

**Table 1:** Demographic and anthropometric data on kidney transplanted children and healthy controls.

	**Transplant** **recipients** ***n*** **= 38**			**Healthy controls** ***n*** **= 17**			***p*-value**
Age (years)	13.6 (7.7–18.0)			11.7 (7.3–18.6)			0.2754
Sex ratio female:male	18:20			7:10			0.67
Height, cm	152 (117–185)			159 (120–185)			
z-score	−0.40 (−2.77–2.63)			1.17 (−0.33–2.01)			<0.0001
Weight, kg	44 (20–75)			50 (21–65)			
z-score	0.08 (−2.56–1.99)			0.28 (−0.80–2.19)			0.5120
BMI, kg/m^2^	19.2 (14.6–30.3)			18.9 (13.1–21.1)			
z-score	0.38 (−2.04–2.39)			−0.57 (−2.37–1.98)			0.0444
SBP, mm Hg	114 (91–134)			103 (85–110)			
z-score	0.64 (−1.35–2.91)			−0.86 (−1.69–0.45)			<0.0001
DBP, mm Hg	70 (50–90)			60 (51–70)			
z-score	0.63 (−1.01–2.54)			−0.27 (−1.23–0.68)			<0.0001
Maximal heart rate, bpm	186 (151–206)			193 (179–208)			0.0107
Minute ventilation, L/min	59.5 (33–108)			76 (28–149)			0.0843
Respiratory exchange ratio	1.15 (1.00–1.38)			1.11 (0.93–1.34)			0.7855
Maximal load, Watt/kg	2.6 (1.6–3.8)			3.5 (2.2–4.4)			
z-score	−1.4 (−3.7–1.4)			0.3 (−1.2–1.5)			<0.0001
VO_2peak_, mL/kg/min	34.5 (18.0–53.0)			43.9 (37.0–50.5)			
z-score	−1.6 (−4.0–1.2)			0.1 (−0.8–0.5)			0.0003
Activity score	1	2	3	1	2	3	
n	19	12	7	0	8	9	0.0003

*BMI, Body mass index; SBP, systolic blood pressure; DBP, diastolic blood pressure. Values presented as Median (range)*.

The underlying kidney diseases were dominated by congenital malformations and hereditary diseases (Table 2). Two patients had been retransplanted before the study start but none of the patients underwent retransplantation during the study period.

**Table 2:** Demographic and biochemical characteristics in kidney transplanted children (*n* = 38).

Age at transplantation, years	9.0 (1.1–16.6)
Time since transplantation, years	3.7 (0.9–13.0)
Gender	
*Male, n*	20
*Female, n*	18
Primary renal disease	
*Congenital malformations CAKUT, n*	14
*Hereditary disease, n*	14
*Acquired disease, n*	9
*Unknown, n*	1
Dialysis time, *n* = 26	0.8 years (4 days-5.4 years)
Donor source	
*Living, n*	29
*Diseased, n*	9
GFR, mL/min/1.73 m^2^	
*3 months post-transplant*	72 (42–112)
*At study*	61 (22–97)
24 h ambulatory blood pressure, z-score	
Day SBP/DBP	0.019/0.228
Night SBP/DBP	−0.282/0.343
FMI, kg/m^2^	5.3 (2.4–14.0)
Laboratory results	
*Hb, g/L*	121 (103–148)
*s-cholesterol, mmol/L*	4.3 (2.9–6.3) (borderline high *n* = 12, high *n* = 2)
*s-HDL, mmol/L*	1.5 (1.0–2.3) (low *n* = 3)
*s-LDL, mmol/L*	2.3 (1.6–4.3) (borderline high *n* = 4, high *n* = 3)
*s-triglycerides, mmol/L*	1.0 (0.5–2.1) (borderline high *n* = 12, high *n* = 6)
Metabolic syndrome criteria	
*BMI z-score >2, n*	2
*Hypertension, n*	25
*s-glucose >5.6 mmol/l, n*	7
*s-triglycerides >1.7 mmol/l, n*	4
*s-HDL cholesterol <1.03 mmol/l, n*	3

*Cut-off for plasma lipids are from the NCEP expert panel on cholesterol levels in children (NHLBI, 2011)*.

*BMI z-score >2, hypertension (systolic or diastolic blood pressure >95th percentile or on antihypertensive treatment), s-glucose >5.6 mmol/L, s-triglycerides >1.7 mmol/L, and s-HDL cholesterol <1.03 mmol/L (Wilson et al., 2010)*.

### Baseline Findings

#### Study Subjects

Immunosuppression was given with prednisolone (30 patients, 24 every other day and six daily), tacrolimus (34 patients) and mycophenolate (21 patients). Three patients received cyclosporine instead of tacrolimus and another three were given everolimus. Anti-hypertensive medication was given to 21 patients, of which 10 had single-therapy calcium blockers, three had single-therapy ACE-inhibitors, three had combination-therapy calcium blockers and beta blockers, and five had combination-therapy calcium blockers and ACE-inhibitors. Median GFR was 61 ml/min/1.73 m^2^. One child had GFR ≥ 90, 19 children had GFR 60–89 (CKD stage 2), 17 children had GFR 30–59 (CKD stage 3) and one child had GFR 15–29 ml/min/1.73 m^2^ (CKD stage 4). All patients had B-Hemoglobin (Hb) >100 g/L. Cholesterol was high in 5%, LDL high in 8%, HDL low in 8% and triglycerides high in 16% (Table 2). No child was given lipid-modifying agents. Two of the patients had exercise induced asthma, and were on beta stimulators before the exercise test.

One of the KT patients had type 2 diabetes mellitus and was considered having metabolic syndrome, meeting 3 of 5 criteria. Ten patients had two criteria, 18 had one and nine patients had no criteria for metabolic syndrome (Table 2).

#### Cross-Sectional Comparisons Between Children With KT and Healthy Controls

The KT patients were significantly shorter than the healthy controls, had higher BMI z-score and higher resting SBP and DBP z-scores. The KT patients showed decreased exercise capacity measured both as VO_2peak_ (34.5 vs. 43.9 ml/kg/min, *p* = 0.0003) and maximal load (2.6 vs. 3.5 W/kg, *p* < 0.0001), also when comparing z-scores (Table 1). No difference was found in RER at peak exercise. There was a significant difference in activity score, where 19 KT patients reported activity score 1, 12 score 2 and 7 score 3 compared to none of the controls reporting score 1, 8 score 2 and 9 score 3, *p* = 0.0003 (Table 1). The median maximum heart rate was lower in the KT patients (186 vs. 193 beats/min, *p* = 0.0107). Three of the KT patients were treated with beta blockers, in two of the patients this likely affected the maximum heart rate, which measured 151, 175, and 201/min in these patients. When comparing controls and KT patients categorized by activity score, there was a trend toward a significantly lower VO_2peak_ z-score in the patients with KT in those with activity score 2 (VO_2peak_ z-score −0.16 and 0.95, *p* = 0.06, *n* = 12 and *n* = 8 for KT patients and controls, respectively) and a significantly lower VO_2peak_ z-score in those with activity score 3 (VO_2peak_ z-score −0.8 and −0.1, *p* = 0.004, *n* = 7 and *n* = 9 for KT patients and controls, respectively).

In multiple regression analysis of both groups combined, the main determinants of VO_2peak_ z-score were activity score and BMI z-score (β = 0.79, *p* < 0.0001 and β = −0.38, *p* = 0.007, respectively).

#### Separate Analysis of Children With KT

LMI and FMI were both associated with BMI in the KT patients (*r* = 0.74, *p* < 0.0001 and *r* = 0.91, *p* < 0.0001, respectively).

**Figure 1:**
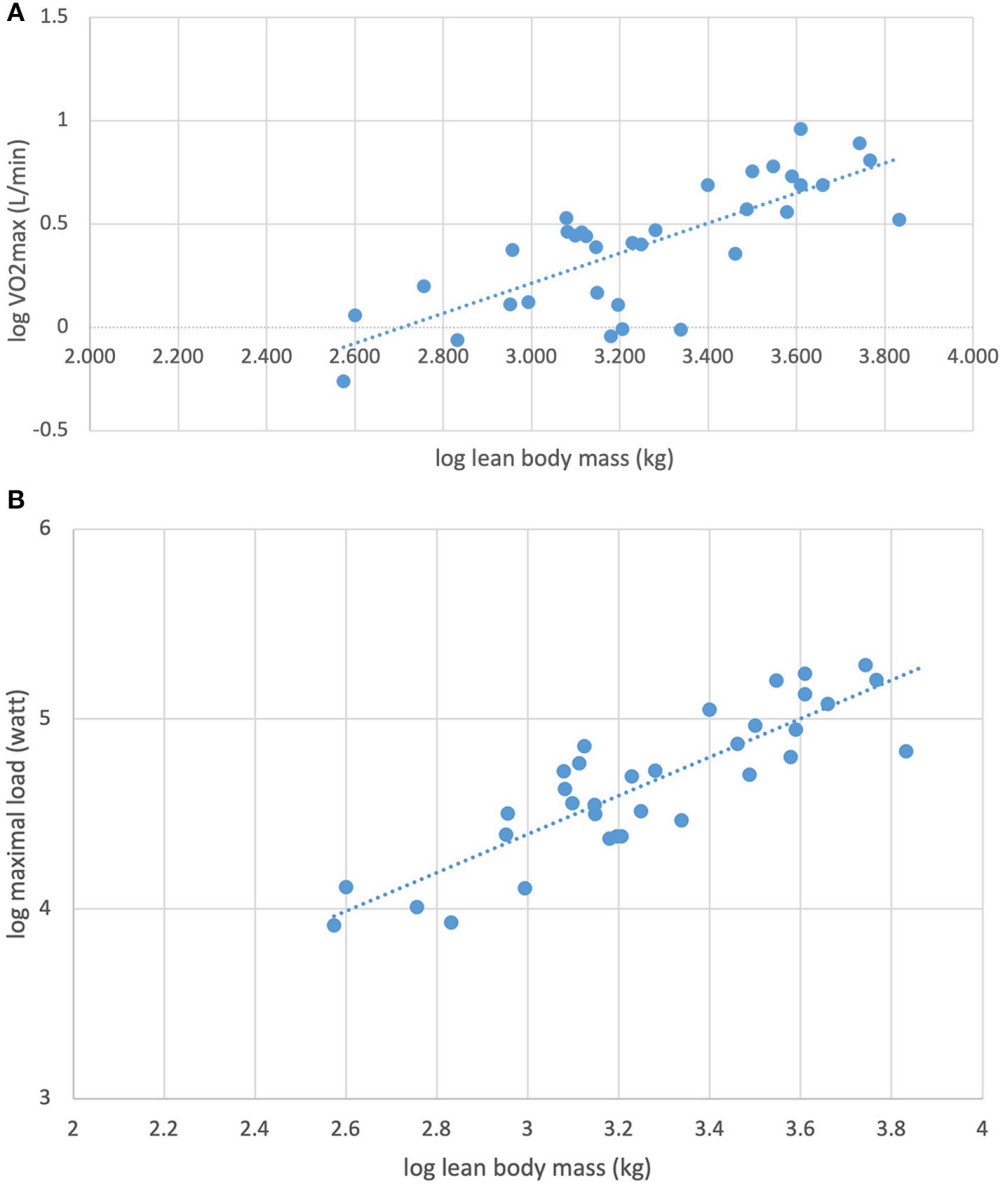
**(A)** Association between log transformed maximal oxygen consumption (VO_2max_) and log transformed lean body mass in 34 kidney transplanted children (*R*^2^ = 0.59, β = 0.73, *p* < 0.0001). **(B)** Association between log transformed maximal load (watt) and log transformed lean body mass in 34 kidney transplanted children (*R*^2^ = 0.75, β = 1.02, *p* < 0.0001).

Linear regression analysis showed that within the KT patient group, VO_2peak_ z-score was determined by FMI, GFR, activity score, LMI, triglycerides, cholesterol and LDL in univariate analyses (Table 3A). No association was found to BMI z-score, systolic and diastolic blood pressure z-score or B-Hb. In a multiple regression model, the main determinants of VO_2peak_ z-score were FMI and GFR. Maximum load z-score was determined by activity score, LMI, LDL and triglycerides but not GFR, blood pressure z-score, cholesterol or BMI z-score (Table 3B). For the KT patients, log transformed VO_2peak_ and maximum load was associated to log transformed lean body mass (Figures 1A,B).

**Table 3A:** Univariable and multivariable linear regression of VO_2_ peak z-score in 38 kidney transplanted children.

	**Univariable linear regression**			**Multivariable linear regression**		
	**β**	**95% CI**	***p*-value**	**β**	**95% CI**	***p*-value**
**VO**_**2**_ **peak z-score**						
FMI, kg/m^2^	−0.37	−0.51, −0.23	<0.0001	−0.36	−0.4945, −0.2329	<0.0001
GFR, mL/min/1.73 m^2^	0.03	0.001, 0.061	0.0430	0.03	0.0054, 0.0510	0.0172
Activity score	0.72	0.19, 1.25	0.0096			n.s.^*^
LMI, kg/m^2^	−0.35	−0.66, −0.05	0.0254			n.s.
Triglycerides	−1.46	−2.42, −0.50	0.0038			n.s
Cholesterol	−0.79	−1.43, −0.14	0.0185			n.s.
LDL-cholesterol	−1.05	−1.76, −0.35	0.0043			n.s.
Hemoglobin	0.02	−0.02, 0.06	0.3370			
Resting SBP z-score	0.41	−0.05, 0.87	0.0771			
Resting DBP z-score	0.17	−0.37, 0.72	0.5182			
BMI z-score	−0.31	−0.73, 0,10	0.1360			

*Multivariable model was selected based on stepwise regression. R^2^ for the multivariable model was 0.47*.

* *Non-significant, not entered in the model*.

**Table 3B:** Univariable and multivariable linear regression of maximal load z-score in 38 kidney transplanted children.

	**Univariable linear regression**			**Multivariable linear regression**		
	**β**	**95% CI**	***p*-value**	**β**	**95% CI**	***p*-value**
**Maximal load z-score**						
FMI, kg/m^2^	−0.34	−0.47, −0.21	<0.0001	−0.29	−0.4158, −0.1608	<0.0001
GFR, mL/min/1.73 m^2^	0.02	−0.0042, 0.0523	0.0934			
Activity score	0.82	0.36, 1.28	0.0009	0.4766	0.06889, 0.88435	0.0234
LMI, kg/m^2^	−0.27	−0.56, 0.02	0.0635			
Triglycerides	−1.06	−1.98, −0.13	0.0267			n.s.^*^
Cholesterol	−0.71	−1.31, −0.11	0.0212			n.s.
LDL-cholesterol	−0.90	−1.55, −0.24	0.0088			n.s.
Hemoglobin	0.002	−0.03, 0.04	0.8980			
Resting SBP z-score	0.28	−0.15, 0.72	0.1935			
Resting DBP z-score	0.25	−0.25, 0.75	0.3228			
BMI z-score	−0.34	−0.72, 0.04	0.0792			

*Multivariable model was selected based on stepwise regression. R^2^ for the multivariable model was 0.48*.

* *Non-significant, not entered in the model*.

ABPM was successfully performed in all 38 children at baseline. Hypertension was found in 12 KT patients (32%) with SBP and/or DBP values above the 95th percentile, eight of whom had antihypertensive treatment. Four of the children with hypertension were classified as non-dippers, and six additional patients were non-dippers. Nine of the hypertensive children had nighttime DBP above the 95th percentile, of whom three also had nighttime SBP and three daytime BP above the 95th percentile. Three children were hypertensive only during daytime. There was no gender difference in day or night blood pressures. There were no correlations between day or night systolic or diastolic blood pressure z-scores and GFR, VO_2peak_ z-score, maximum load z-score, BMI z-score or lipid levels.

Echocardiography was performed in 28 KT patients at baseline. Median LVMI was 31.4 g/m^2,7^ (range 20.8–45.2), LVM 98.3 g (51.7–206.2), LVd 4.5 cm (3.9–6.0), IVSd 7.0 mm (4.9–10.0), and PWd 6.7 mm (4.4–9.5). Three patients (10%) had LVMI above 95th percentile for height. There was no correlation between LVMI and blood pressure z-scores.

### Longitudinal Data

**Figure 2:**
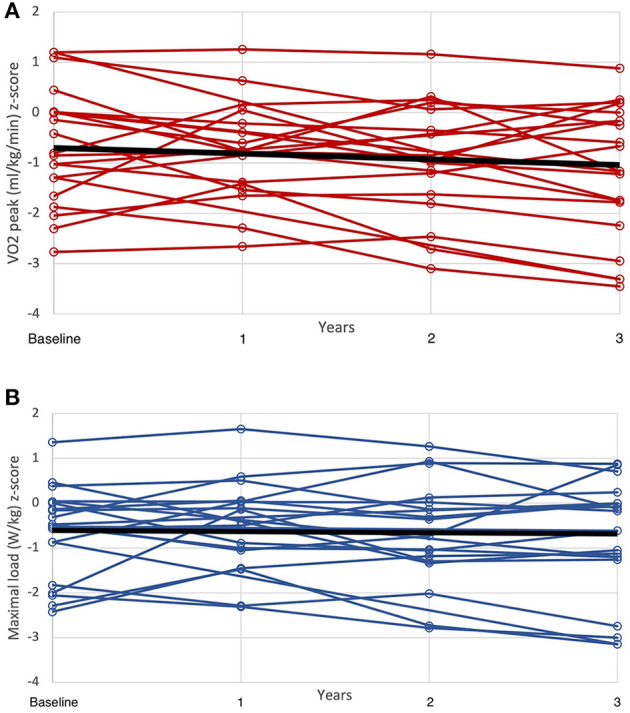
**(A)** Development of maximal oxygen consumption (VO_2max_/kg z-score) during the 3-year follow-up. **(B)** Development of maximal load (watt/kg z-score) during the 3-year follow-up.

The longitudinal data were captured for 1, 2, and 3 years in 33, 22, and 17 individuals, respectively. The KT patients showed no change in exercise capacity z-scores over time (Figures 2A,B). Median GFR after 3 years was 60 mL/min/1.73 m^2^ (range 35–88). There was an increase in night systolic blood pressure by 0.66 z-scores (median, range −1.71–3.01) after 3 years but no significant change in day blood pressures or night diastolic blood pressure. There was no correlation between blood pressure changes and GFR at start or after 3 years.

## Discussion

We found a decreased exercise capacity and increased BP in children with KT compared to matched healthy controls. The exercise capacity was predicted by high activity score and low BMI z-score in the groups combined. In the multivariable logistic regression model within the KT-cohort, the main predictors for impaired exercise capacity were FMI, activity score and GFR (Table 3).

Exercise capacity in general is dependent on age, sex and cardiovascular function, and by using the z-score for VO_2peak_ and maximal load derived from Ten Harkel et al. (2011) we could reduce the impact of age and sex. The exercise capacity also depends on muscle mass, metabolic factors, activity level and cardiac function, all of which can be affected by disease severity, medication and other disease related factors in children with KT.

### Disease Severity and Activity Level

Baseline GFR was a significant determinant of VO_2peak_ z-score in the children with KT, suggesting that there is a significant impact by disease severity. This could be multi-factorial, as severe CKD and its treatment affects muscle mass (Alayli et al., 2008), fat mass and cardiac function. It is also a reflection of the inactivity seen in many children with CKD, before as well as after transplantation (Painter et al., 2007; Clark et al., 2016).

Our general practice is to perform preemptive transplantation if possible. When dialysis is unavoidable, automated peritoneal dialysis (PD) is preferred. None of the 38 children with KT in the study were on dialysis and the median time in dialysis before transplantation was relatively short. Nevertheless, children on PD have been shown to have a reduced exercise capacity, mainly due to decreased muscle strength (Alayli et al., 2008).

Chronic kidney disease and the need for dialysis may limit the level of activity due to low levels of physical and mental energy. Neurocognitive problems and concentration difficulties are seen in this group (Holmberg and Jalanko, 2016) and this could contribute to the lower exercise tolerance often seen in these patients (Master Sankar Raj et al., 2017). Children with CKD are in general more sedentary than their healthy peers (Clark et al., 2016) and they are hard to motivate for physical activities (van Bergen et al., 2009). Transplant recipients commonly have a fear of kidney injury from participating in contact sport and ball sport, and they are often recommended to limit sports participation (Wolf et al., 2016) and to avoid contact sports (Master Sankar Raj et al., 2017). Parental overprotection is also common in families with chronically ill children (Hullmann et al., 2010). These factors may contribute to the lower activity score seen in our KT patients, 18 of them with CKD 3-4. Of the KT patients, 50% had no regular physical activity, and moderate to vigorous activity for 3 h per week or more was seen more frequently in the control group. However, when comparing groups with similar activity level, the KT patients still had a lower exercise capacity, suggesting an impact on both activity level and exercise capacity. This is supported by similar findings in a Norwegian study in children after KT where 19 of 22 children reported <60 min of moderate to vigorous daily physical activity (Tangeraas et al., 2010).

### Muscle Mass and Body Composition

There are different physiological aspects of cardiopulmonary exercise capacity, and one major determinant is the muscle mass, where most of the oxygen is consumed during exercise. As BMI do not properly reflect the muscle mass, we used the assessment of body composition by DXA in order to better determine this impact. In our study, we have used LMI as a proxy for muscle mass in the children with KT. In the linear regression analysis, LMI was associated with both VO_2peak_ and maximal load, but did not remain an independent determinant in the multiple regression analysis. When analyzing log transformation of both VO_2peak_ and maximal load, we did find a strong association with log transformed lean body mass, in accordance to the results from the study by Sethna et al. (2009) (Figures 1A,B).

In our multiple regression analysis, the main determinant for exercise capacity was fat mass, measured as FMI. This may explain part of the decreased VO_2peak_, since one major side effect of immunosuppressants used in KT patients is increased body fat, often coupled with a decreased lean body mass (muscle mass). Indications of this difference between control subjects and KT patients were found in the difference in BMI z-score. However, this could not be confirmed in our study, as DXA was only performed in the children with KT. BMI z-score in this age group has its limitations, as it does not properly reflect body composition, and may lead to misclassification of overweight and obesity (Dangardt et al., 2019). A greater muscle mass in control subjects would also increase their BMI z-score. This could mask the difference in body composition between groups, as well as contribute to an increased exercise capacity in the controls. However, as there was a strong negative correlation between BMI z-score and VO_2peak_ z-score in the control group, this may not be the case in our cohort. In addition, FMI was strongly correlated to BMI in the KT children, and more so than LMI, indicating that FMI is a main determinant of BMI in this group. This was furthermore supported by the unexpected negative prediction of lean body mass index (LMI) for VO2peak in the univariate analysis, as opposed to the positive correlation between lean body mass and VO2peak. One reason for this may be that obesity is often associated with an increased muscle mass and an increased LMI, without the concurrent increase in exercise capacity otherwise seen in non-obese subjects with increased muscle mass.

### Metabolic Syndrome and Risk Factors

Children with KT are at risk for developing metabolic risk factors and metabolic syndrome, often during the first year post-transplantation (Wilson et al., 2010). Since our patients had a median time post transplantation of 3.7 years (0.9–13.0) we would expect most metabolic risk factors to have emerged. In our study, we used a combination of three or more risk factors to define metabolic syndrome in accordance to the study by Wilson et al. (2010). However, even with only one patient fulfilling three of the five criteria for metabolic syndrome, 76% of the patients still had isolated dyslipidemia, hypertension or obesity, or a combination of two criteria. The association between exercise capacity and metabolic factors is not completely clear. However, we know that impaired cardio-respiratory fitness may be caused by decreased mitochondrial function and increased intramyocellular fat, as shown in obese patients by Weiss et al. (2003). In our KT patients, high triglycerides, LDL and total cholesterol were all associated with decreased VO_2peak_ z-score, suggesting a decreased exercise capacity with dyslipidemia. This may in part be a proxy for increased fat mass, which is indicated by the association with BMI z-score we found. It is also supported by the strong association between FMI and BMI in this group. However, only a few patients had dyslipidemia or obesity in our cohort using the Wilson criteria (Wilson et al., 2010), but using the stricter criteria from Habbig et al. (2017) 76% of the KT children would have been classified as having dyslipidemia at baseline.

Another metabolic factor that may affect the VO_2peak_ z-score is the oxygen transporter hemoglobin (Hb). In our cohort, the median Hb was 121 g/L, and we did not find any patient with Hb <100 g/L, suggesting that anemia was not an important factor decreasing oxygen consumption during exercise in our cohort. We did not find any association between VO_2peak_ z-score and Hb, supporting this finding.

The most common risk factor in our cohort was hypertension, frequently found in patients with KT (Charnaya and Moudgil, 2017). We did not find any association between any of the blood pressure measures and exercise capacity, implicating that this is not a major limiting factor for VO_2peak_ z-score or maximal load z-score.

In an American review of 234 children with KT by Wilson et al., 45.3% of the children were overweight or obese (BMI >85th percentile) and 37.6% met the criteria for metabolic syndrome (Wilson et al., 2010). The children in our study were less overweight and had less abnormal metabolic factors with only 9 patients (24%) with BMI >85th percentile and one patient fulfilling the criteria for the metabolic syndrome. Nevertheless, we found a similar decrease in exercise capacity as others have shown in similar populations (Painter et al., 2007; Weaver et al., 2008; van Bergen et al., 2009), perhaps suggesting that the metabolic risk factors are not determinant for exercise capacity in these patients.

### Cardiac and Respiratory Function

Other important physiological factors influencing exercise capacity are cardiac function and lung function.

The cardiac function during exercise is mainly determined by cardiac output, which is a function of the heart rate and the stroke volume. The maximal heart rate is affected by hormones, autonomic function, and fitness level. The stroke volume is dependent mainly on cardiac contractility, vascular resistance, fitness level and ventricular size. In our patients, even though only three had betablockers, there was a 4% decreased maximal heart rate as compared to controls. This may indicate a chronotropic limitation causing decreased exercise capacity, as a decreased maximal heart rate leads to a decreased cardiac output. Only three patients showed signs of LV hypertrophy, indicating affected cardiac function and, subsequently, a possible impact on stroke volume and diastolic function, as has been suggested by the study by Weaver and colleagues (Weaver et al., 2008).

Regarding the respiratory function, there was no significant difference in minute ventilation at peak exercise, suggesting that there was no respiratory limitation explaining the decreased exercise capacity in the children with KT. This is supported by the finding of Feber et al. who found normal vital capacity and forced expiratory volume in 1 s in most of the patients in their study on physical performance in children after kidney transplantation (Feber et al., 1997).

### Ambulatory Blood Pressure Monitoring

The prevalence of hypertension in our cohort was 12%. Most of these patients had elevated nighttime diastolic BP and only 3 patients had an isolated daytime hypertension. This high proportion of masked hypertension underscores the importance of ABPM to evaluate the blood pressure in children after KT, known to be at risk of future cardiovascular events. It has also been shown that night time blood pressure is a better predictor of cardiovascular outcome in hypertensive adult patients (Fagard et al., 2008).

The increased BP was not associated with increased LVMI in our patients, contrary to the study by Wilson and colleagues, where they found an association between high BP and increased LVMI. Their higher prevalence of metabolic syndrome and overweight/obesity may at least partly explain these differences (Wilson et al., 2010).

### Strengths and Limitations

The main strength of this study was the complete enrollment of all eligible patients at our center, only excluding patients that were physically unable to perform the exercise test. All investigations used are well-established. They were conducted by the same investigators in all patients, and one investigator (TL) did all the semi structured interviews for activity scoring.

As this was an observational study of the cohort of KT children at our center, with a cross sectional design, the underlying kidney disease varied between the patients. They also had different duration of kidney failure and time in dialysis before transplantation and varying time interval between transplantation and inclusion in the study. Also, DXA and biochemistry data, ABPM data, as well as longitudinal data on exercise capacity, were not collected in the control group. The cardiac ultrasound had to be excluded in 11 children, as it was not performed in conjunction with the exercise test.

The controls were fewer than the KT children. The groups were matched for age and sex ratio, but not on an individual basis. This is a weakness, potentially adding bias to the study. Despite the low number of children in the KT and control groups, we had significant results in many of the analyses, confirming the results from other studies.

### Conclusion

In this group of patients with KT showed decreased exercise capacity and increased BP as compared to age and gender-matched healthy controls. The lower exercise capacity could be explained by lower activity score, decreased GFR and high fat mass. The exercise capacity in patients with KT did not improve over time.

### Future Implications

The decreased exercise capacity in patients with KT did not improve over time. There were no intervention in terms of exercise program or the likes involved in the standard care program for these patients. Considering the results from this study, this may be a future priority, which potentially could improve not only the cardiorespiratory fitness, but also the metabolic state of these patients.

## Data Availability Statement

The raw data supporting the conclusions of this article will be made available by the authors, without undue reservation.

## Ethics Statement

The studies involving human participants were reviewed and approved by Regionala Etikprovningsnamnden in Gothenburg Medical Division Gothenburg, SWEDEN. Written informed consent to participate in this study was provided by the participants' legal guardian/next of kin.

## Author Contributions

SW, FD, SH, and TL were responsible for the planning and design of the study. TL, FD, SW, PB, and SH performed the examinations. EB, OH, and JK assisted in analyzing data. All authors assisted in interpreting the findings and writing the manuscript.

## Conflict of Interest

The authors declare that the research was conducted in the absence of any commercial or financial relationships that could be construed as a potential conflict of interest.

## Publisher's Note

All claims expressed in this article are solely those of the authors and do not necessarily represent those of their affiliated organizations, or those of the publisher, the editors and the reviewers. Any product that may be evaluated in this article, or claim that may be made by its manufacturer, is not guaranteed or endorsed by the publisher.
